# Clinical manifestations of focal segmental glomerulosclerosis in Japan from the Japan Renal Biopsy Registry: age stratification and comparison with minimal change disease

**DOI:** 10.1038/s41598-020-80931-9

**Published:** 2021-01-28

**Authors:** Takaya Ozeki, Shoichi Maruyama, Toshiyuki Imasawa, Takehiko Kawaguchi, Hiroshi Kitamura, Moritoshi Kadomura, Ritsuko Katafuchi, Kazumasa Oka, Hitoshi Yokoyama, Hitoshi Sugiyama, Hiroshi Sato

**Affiliations:** 1 Department of Nephrology, Nagoya University Graduate School of Medicine, 65 Tsurumai-cho, Showa-ku, Nagoya, 466-8550 Japan; 2 Department of Nephrology, National Hospital Organization Chibahigashi National Hospital, Chiba, Japan; 3 Department of Pathology, National Hospital Organization Chibahigashi National Hospital, Chiba, Japan; 4 Department of Nephrology, Medical Corporation Houshikai Kano Hospital, Fukuoka, Japan; 5 Kidney Unit, National Hospital Organization Fukuoka Higashi Medical Center, Fukuoka, Japan; 6 Department of Pathology, Hyogo Prefectural Nishinomiya Hospital, Hyogo, Japan; 7 Department of Nephrology, Kanazawa Medical University School of Medicine, Ishikawa, Japan; 8 Department of Human Resource Development of Dialysis Therapy for Kidney Disease, Okayama University Graduate School of Medicine, Dentistry and Pharmaceutical Sciences, Okayama, Japan; 9 Department of Internal Medicine, Sendai Hospital of East Japan Railway Company, Sendai, Japan

**Keywords:** Medical research, Nephrology

## Abstract

Focal segmental glomerulosclerosis (FSGS) is a serious condition leading to kidney failure. We aimed to investigate the clinical characteristics of FSGS and its differences compared with minimal change disease (MCD) using cross-sectional data from the Japan Renal Biopsy Registry. In Analysis 1, primary FSGS (n = 996) were stratified by age into three groups: pediatric (< 18 years), adult (18–64 years), and elderly (≥ 65 years), and clinical characteristics were compared. Clinical diagnosis of nephrotic syndrome (NS) was given to 73.5% (97/132) of the pediatric, 41.2% (256/622) of the adult, and 65.7% (159/242) of the elderly group. In Analysis 2, primary FSGS (n = 306) and MCD (n = 1303) whose clinical diagnosis was nephrotic syndrome (NS) and laboratory data were consistent with NS, were enrolled. Logistic regression analysis was conducted to elucidate the variables which can distinguish FSGS from MCD. On multivariable analysis, higher systolic blood pressure, higher serum albumin, lower eGFR, and presence of hematuria associated with FSGS. In Japanese nationwide registry, primary FSGS patients aged 18–64 years showed lower rate of NS than those in other ages. Among primary nephrotic cases, FSGS showed distinct clinical features from MCD.

## Introduction

Focal segmental glomerulosclerosis (FSGS) presents with proteinuria, often accompanied by nephrotic syndrome (NS), and may lead to end-stage renal disease^1^. FSGS originally referred to steroid-resistant nephrotic syndrome in pediatric patients with segmental obliteration of glomerular capillaries, with or without hyalinosis on light microscopy^2^. The entity of FSGS has been expanded and FSGS is currently regarded as a group of kidney diseases sharing common glomerular lesions^3^. There are two pathophysiological types of FSGS: primary and secondary ones. In primary FSGS, it is postulated that circulating permeability factors cause podocyte injury, inducing NS^3,4^. Whereas, secondary cases have underlying etiologies such as hypertension, obesity, viruses, drugs, genetic mutations, or adaptive conditions^4,5^, and they do not always manifest NS. Therefore, the clinical presentations of FSGS are diverse. Cases with NS (usually the primary form) show a different clinical course compared with minimal change disease (MCD) i.e. poor therapeutic response, worse renal prognosis^1,6^, and rapid recurrence of proteinuria after kidney transplantation^7^. However, it is difficult to clearly distinguish nephrotic FSGS from MCD because of their similar clinical presentations and the sampling limitations of pathological specimens^8,9^. To date, few studies have described the clinical features of FSGS, as well as the distinction between nephrotic FSGS and MCD using a large sample size.

The Japan Renal Biopsy Registry (J-RBR) is a nationwide, web-based, registry of renal biopsies established by the Japanese Society of Nephrology in 2007^10^. Cross-sectional studies using this database have described the clinical features of several kidney diseases in Japan^11–19^.

The present study aimed to clarify the clinical characteristics of FSGS patients in Japan by age stratification of them and comparison with MCD patients through an analysis of data from the J-RBR compiled between July 2007 and June 2016.

## Results

**Figure 1:**
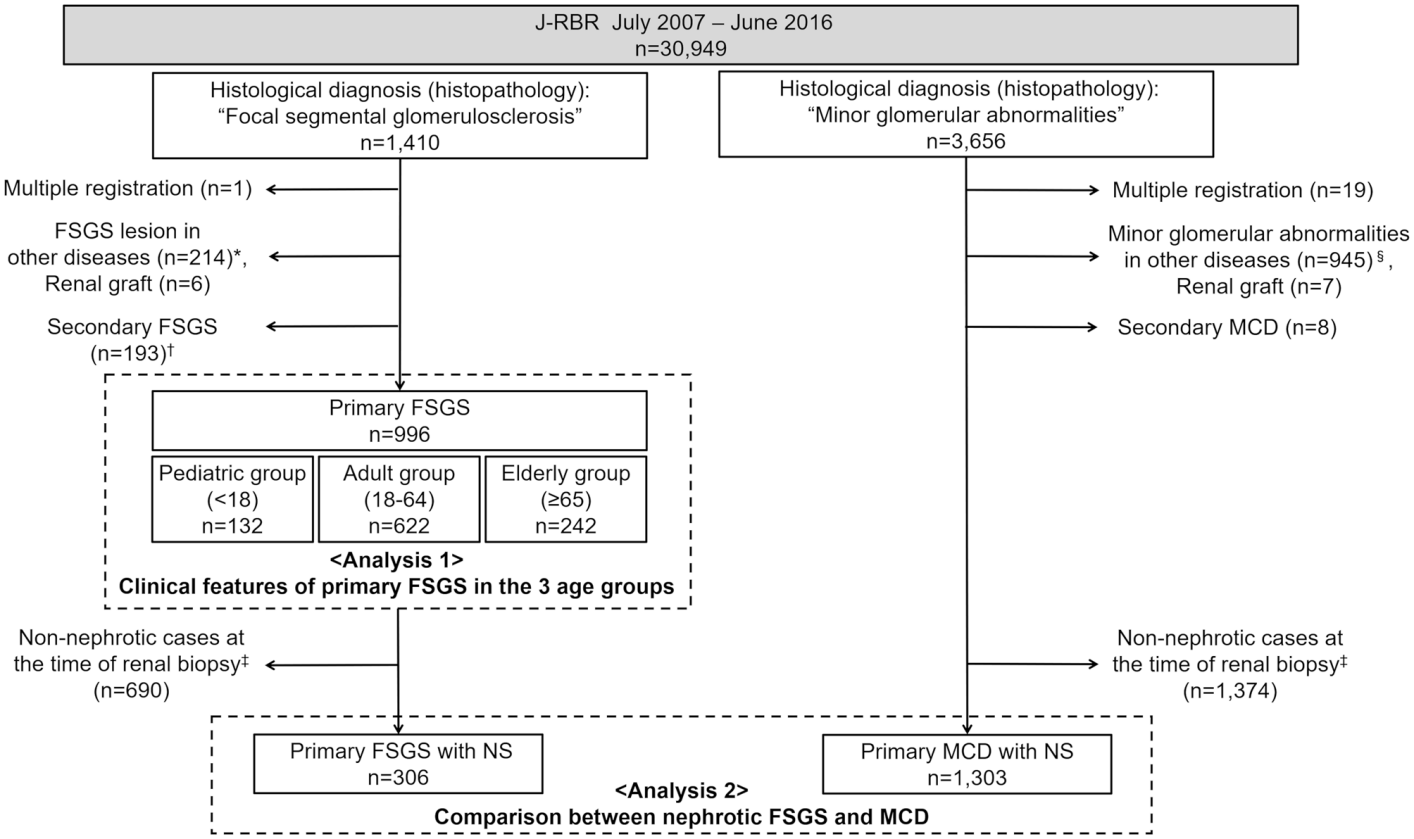
Flow of patient selection in present study. *The details of distribution of histopathologically diagnosed “focal segmental glomerulosclerosis” including FSGS lesion in other diseases are shown in Supplementary Fig. S1 and Supplementary Table S1. ^†^The details of secondary FSGS are shown in Supplementary Fig. S3. ^‡^Nephrotic cases at the time of renal biopsy was defined as the cases whose clinical diagnosis was nephrotic syndrome and who fitted the laboratory criteria of nephrotic syndrome. Laboratory criteria for nephrotic syndrome, for pediatric patients (age < 18): urinary protein ≥ 40 mg/h/m^2^ or ≥ 2.0 g/gCr and serum albumin ≤ 2.5 g/dL; for adult and elderly patients (age ≥ 18): urinary protein ≥ 3.5 g/day or ≥ 3.5 g/gCr and serum albumin ≤ 3.0 g/dL. ^§^The details of distribution of histopathologically diagnosed “minor glomerular abnormalities” including minor glomerular abnormalities in other diseases are shown in Supplementary Fig. S2 and Supplementary Table S2. *J-RBR* Japan Renal Biopsy Registry, *FSGS* focal segmental glomerulosclerosis, *MCD* minimal change disease, *NS* nephrotic syndrome.

Among 30,949 patients who registered to J-RBR between July 2007 and June 2016, 1,410 cases were extracted for the evaluation of FSGS. Additionally, 3656 patients were also extracted for the evaluation of MCD (Fig. 1).

### General demographics of FSGS in the J-RBR

**Figure 2:**
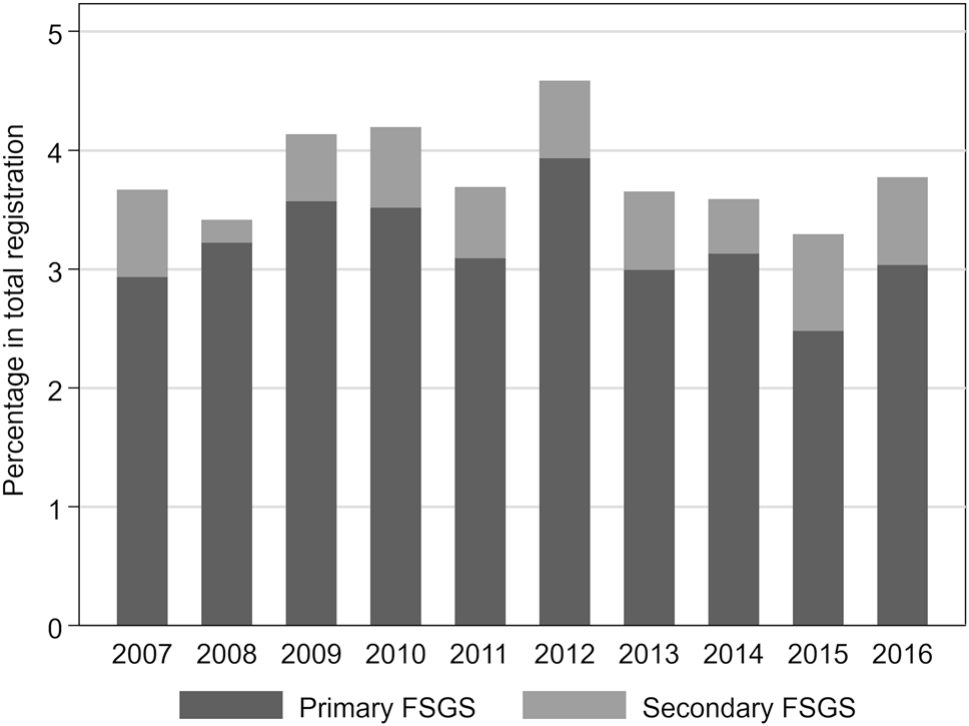
Annual incidence of FSGS in the J-RBR. *J-RBR* Japan Renal Biopsy Registry, *FSGS* focal segmental glomerulosclerosis.

After removing a patient with multiple registration, 1409 patients were registered as histologically registered as focal segmental glomerulosclerosis in J-RBR. Of these, 996 were primary FSGS and 193 were secondary FSGS (Supplementary Fig. S1). The annual incidence of FSGS accounted for 3.5–4.5% of all registered cases in the J-RBR database. The percentage of primary FSGS were constant at approximately 3% of all during 2007 to 2016 (Fig. 2). The details of secondary FSGS were summarized in Supplementary Fig. S3.

### Analysis 1: description of clinical features of primary FSGS in the three age groups

For Analysis 1, 996 primary FSGS cases were divided into three age groups: pediatric group (n = 132), adult group (n = 622), and elderly group (n = 242). The detailed age-stratified distribution of the prevalence and proportion of patients with FSGS and MCD are shown in Supplementary Fig. S4. The clinical features of the patients are summarized in Table 1. Patients who underwent biopsy more than twice were the most common in the pediatric group (26.5%). In clinical diagnosis, NS was the most common (51.4%), followed by chronic nephritic syndrome (44.4%) in total. A clinical diagnosis of NS was 73.5% in the pediatric group, 41.2% in the adult group, and 65.7% in the elderly group. In the adult group, prevalence of chronic nephritic syndrome (54.3%) was higher than that of NS. Body mass index was greater and kidney function was worse in the adult and elderly groups than in the pediatric group (P < 0.001). The elderly group showed the highest systolic blood pressure and highest prevalence of concomitant hypertension on antihypertensive drugs (68.8%) and diabetes mellitus (18.8%). Baseline estimated glomerular filtration rate (eGFR) was highest in the pediatric group, followed by the adult group and elderly group (eGFR: 106, 64, and 40 mL/min/1.73 m^2^, respectively). Fewer patients who were consistent with NS in laboratory data at biopsy were found in the pediatric group and adult group than in the elderly group (24.2%, 27.5%, and 51.2%, respectively).

**Table 1:** Clinical features of primary FSGS in the Japan Renal Biopsy Registry.

	Obs	Overall (n = 996)		Age groups						P-value
				Pediatric group (n = 132)		Adult group (n = 622)		Elderly group (n = 242)		
**Patient characteristics**										
Age	996	47	[89–64]	10	[4–15]	43	[32–55]	72	[68–77]	< 0.001
Sex (male)	996	592	(59.4)	80	(60.6)	360	(57.9)	152	(62.8)	0.40
Number of biopsies										
First	996	516	(51.8)	52	(39.4)	322	(51.8)	142	(58.7)	< 0.001
Second		66	(6.6)	24	(18.2)	33	(5.3)	9	(3.7)	
≥ 3 times		26	(2.6)	11	(8.3)	14	(2.6)	1	(0.4)	
Unknown		388	(39.0)	45	(34.1)	253	(40.7)	90	(37.2)	
Clinical diagnosis										
Nephrotic syndrome	996	512	(51.4)	97	(73.5)	256	(41.2)	159	(65.7)	< 0.001
Chronic nephritic syndrome		442	(44.4)	31	(25.5)	338	(54.3)	73	(30.2)	
Others		42	(4.2)	4	(3.0)	28	(4.5)	10	(4.1)	
Body Mass Index	982	22.9	[20.3–26.2]	18.5	[16.3–21.5]	23.5	[20.8–27.1]	23.6	[21.4–25.9]	< 0.001
Systolic blood pressure	828	129	[118–142]	113	[103–121]	129	[119–141]	138	[125–150]	< 0.001
Diastolic blood pressure	828	78	[68–86]	68	[60–78]	80	[70–88]	77	[70–86]	< 0.001
Antihypertensive drugs	817	414	(50.7)	31	(34.1)	244	(46.6)	139	(68.8)	< 0.001
Diabetes mellitus	717	99	(13.8)	4	(4.8)	61	(13.5)	34	(18.8)	0.013
**Laboratory data**										
Total protein, g/dL	988	6.2	[4.9–7.0]	6.1	[5.0–6.9]	6.5	[5.0–7.0]	5.5	[4.8–6.6]	< 0.001
Albumin, g/dL	986	3.5	[2.2–4.1]	3.6	[2.6–4.2]	3.7	[2.3–4.1]	2.6	[2.0–3.75	< 0.001
Total cholesterol, mg/dL	975	235	[195–323]	250	[177–399]	232	[195–308]	242	[195–325]	0.53
HbA1c (NGSP), %	608	5.7	[5.4–6.0]	5.5	[5.3–5.8]	5.6	[5.4–6.0]	5.8	[5.4–6.0]	0.043
Creatinine, mg/dL	994	0.96	[0.70–1.31]	0.44	[0.31–0.66]	0.95	[0.73–1.26]	1.24	[0.93–1.69]	< 0.001
eGFR, mL/min/1.73 m^2^	987	60	[41–84]	106	[83–134]	64	[47–83]	40	[29–57]	< 0.001
Urinary protein, g/gCr	697	2.9	[1.02–6.93]	1.86	[0.16–6.16]	2.27	[0.98–5.46]	5.66	[2.83–9.70]	< 0.001
Urinary protein, g/day	680	2.24	[0.84–5.49]	1.65	[0.17–6.80]	1.96	[0.84–5.01]	3.68	[1.24–6.44]	< 0.001
Consistent with NS^a^	996	327	(32.8)	32	(24.2)	171	(27.5)	124	(51.2)	< 0.001
Urinary occult blood										
(−)	996	323	(32.4)	70	(53.0)	205	(33.0)	48	(19.8)	< 0.001
(±)		143	(14.4)	14	(10.6)	94	(15.1	35	(14.5)	
(1+)		170	(17.1)	10	(7.6)	100	(16.1	60	(24.8)	
(2+)		208	(20.9)	20	(15.2)	129	(20.7)	59	(24.4)	
(3+)		152	(15.3)	18	(13.6)	94	(15.1)	40	(16.5)	
Urinary occult blood present^b^	996	530	(53.2)	48	(36.4)	323	(51.9)	159	(65.7)	< 0.001
Urinary RBC/HFP										
(−)	996	154	(15.5)	32	(24.2	94	(15.1)	28	(11.6)	0.025
< 5		448	(45.0)	49	(37.1)	287	(46.1)	112	(46.3)	
5–10		163	(16.4)	18	(13.6)	97	(15.6)	48	(19.8)	
10–30		129	(13.0)	16	(12.1)	77	(12.4)	36	(14.9)	
Many		102	(10.2)	17	(12.9)	67	(10.8)	18	(7.4)	
Urinary RBC present^c^	996	394	(39.6)	51	(38.6)	241	(38.8)	102	(42.2)	0.64
Hematuria present^d^	996	363	(36.5)	41	(31.1)	226	(36.3)	96	(39.7)	0.26

Data are presented as median [interquartile range] for continuous variables and count (percentage) for categorical variables.

*obs* number of observations, *eGFR* estimated glomerular filtration rate, *NGSP* National Glycohemoglobin Standardization Program, *NS* nephrotic syndrome, *RBC* red blood cell, *HPF* high powered field.

^a^Laboratory criteria for nephrotic syndrome, for pediatric patients (age < 18): urinary protein ≥ 40 mg/h/m^2^ or ≥ 2.0 g/gCr and serum albumin ≤ 2.5 g/dL; for adult and elderly patients (age ≥ 18): urinary protein ≥ 3.5 g/day or ≥ 3.5 g/gCr and serum albumin ≤ 3.0 g/dL.

^b^Urinary occult blood present, (1+), (2+), (3+) on dipstick.

^c^Urinary RBC present, ≥ 5/HPF in urine sediment.

^d^Hematuria present, (1+), (2+), (3+) on dipstick and ≥ 5/HPF in sediment.

### Analysis 2: comparison between nephrotic FSGS and MCD

Of 1410 patients with “focal segmental glomerulosclerosis” and 3656 with “minor glomerular abnormalities” on histological diagnosis (histopathology), 306 with nephrotic FSGS and 1303 with MCD were included in Analysis 2 (Fig. 1). The characteristics of nephrotic FSGS and MCD at the biopsy are summarized in Table 2. FSGS cases were older (FSGS: 58 vs. MCD: 44 years), had a higher prevalence of hypertension on antihypertensive drugs (56.2 vs. 28.7%), and lower eGFR (53 vs. 72 mL/min/1.73 m^2^). FSGS patients also had higher serum albumin (1.9 vs. 1.7 g/dL), lower daily urinary protein levels (6.28 vs. 7.00 g/day) and higher prevalence of hematuria (52.9 vs. 29.8%) at biopsy.

**Table 2:** Comparison between nephrotic FSGS and MCD cases.

	Nephrotic FSGS			MCD			P-value
	(n = 306)			(n = 1303)			
	Obs	Median, n	[IQR], (%)	Obs	Median, n	[IQR], (%)	
**Patient characteristics**							
Age	306	58	[34–71]	1303	44	[26–66]	< 0.001
Sex (male)	306	184	(60.1)	1303	749	(57.5)	0.40
Body Mass Index	305	22.9	[20.5–26.1]	1296	23.4	[20.8–26.2]	0.41
Systolic blood pressure	265	136	[121–148]	1125	123	[111–135]	< 0.001
Diastolic blood pressure	265	80	[70–90]	1125	74	[66–83]	< 0.001
Antihypertensive drugs	242	136	(56.2)	1087	312	(28.7)	< 0.001
Diabetes mellitus	215	33	(15.4)	971	131	(13.5)	0.48
**Laboratory data**							
Total protein, g/dL	304	4.7	[4.1–5.3]	1299	4.5	[4.0–5.0]	< 0.001
Albumin, g/dL	306	1.9	[1.5–2.4]	1303	1.7	[1.3–2.1]	< 0.001
Total cholesterol, mg/dL	302	348	[275–430]	1297	411	[328–499]	< 0.001
HbA1c (NGSP), %	204	5.6	[5.3–6.0]	887	5.6	[5.4–6.0]	0.32
Creatinine, mg/dL	304	1.03	[0.75–1.64]	1302	0.85	[0.64–1.11]	< 0.001
eGFR, mL/min/1.73 m^2^	302	53	[32–73]	1289	72	[51–90]	< 0.001
Urinary protein, g/gCr	243	8.17	[5.50–11.00]	1042	8.47	[5.50–11.92]	0.24
Urinary protein, g/day	232	6.28	[4.54–8.87]	983	7.00	[4.75–10.20]	0.015
Urinary occult blood							
(−)	306	27	(8.8)	1303	258	(19.8)	< 0.001
(±)		40	(13.1)		221	(17.0)	
(1+)		82	(26.8)		272	(20.9)	
(2+)		104	(34.0)		403	(30.9)	
(3+)		53	(17.3)		149	(11.4)	
Urinary occult blood present*	306	239	(78.1)	1303	824	(63.2)	< 0.001
Urinary RBC/HPF							
(−)	306	23	(7.5)	1303	201	(15.4)	< 0.001
< 5		114	(37.3)		688	(52.8)	
5–10		81	(26.5)		214	(16.4)	
10–30		56	(18.3)		142	(10.9)	
Many		32	(10.5)		58	(4.5)	
Urinary RBC present^a^	306	169	(55.2)	1303	414	(31.8)	< 0.001
Hematuria present^a^	306	162	(52.9)	1303	388	(29.8)	< 0.001

*FSGS* focal segmental glomerulosclerosis, *MCD* minimal change disease, *eGFR* estimated glomerular filtration rate, *RBC* red blood cell, *HPF* high powered field.

^a^Definition: Urinary occult blood present, (1+), (2+), (3+) on dipstick; Urinary RBC present, ≥ 5/HPF in urine sediment; Hematuria present, (1+), (2+), (3+) on dipstick and ≥ 5/HPF in sediment.

As summarized in Table 3, univariate logistic regression analysis revealed that age, systolic blood pressure, serum albumin, serum total cholesterol, serum creatinine (log-transformation), eGFR and presence of urinary red blood cells and hematuria were significantly associated with a diagnosis of FSGS. The correlations between continuous variables are shown in Supplementary Table S3. Patient age, systolic blood pressure, serum albumin, eGFR, daily urinary protein level (log-transformation), and presence of hematuria were used in the multivariate model (Table 3). Because of the strong correlation between patient age and eGFR, we evaluated two models that took one of them: Model 1 and Model 2. In Model 1, FSGS was associated with higher systolic blood pressure (OR 1.25, 95% confidence interval [CI] 1.14–1.36, for every increase of 10 mmHg), higher serum albumin (OR 2.13, 95% CI 1.56–2.91), lower eGFR (OR 0.90, 95% CI 0.85–0.95, every increase of 10 mL/min/1.73m^2^), and presence of hematuria (OR 1.92, 95% CI 1.36–2.69). In Model 2, higher systolic blood pressure (OR 1.31, 95% CI 1.19–1.44, for every increase of 10 mmHg), higher serum albumin (OR 2.02, 95% CI 1.49–2.73), and presence of hematuria (OR 2.18, 95% CI 1.56–3.04).

**Table 3:** Associating factors with FSGS vs. MCD.

Variables	Univariate			Multivariable					
				Model 1^b^			Model 2^c^		
	OR	[95% CI]	P-value	OR	[95% CI]	P-value	OR	[95% CI]	P-value
Age (every 10 years)	1.13	[1.07–1.20]	< 0.001				0.98	[0.90–1.07]	0.72
Sex (male)	1.12	[0.87–1.44]	0.40						
Body Mass Index	1.00	[0.98–1.03]	0.82						
Systolic BP (every 10 mmHg)	1.38	[1.28–1.48]	< 0.001	1.25	[1.14–1.36]	< 0.001	1.31	[1.19–1.44]	< 0.001
Anti-hypertensive drugs	3.19	[2.39–4.24]	< 0.001						
Diabetes mellitus	1.16	[0.77–1.76]	0.48						
HbA1c (NGSP)	0.86	[0.68–1.09]	0.21						
Total protein	1.11	[0.99–1.22]	0.066						
Albumin	2.24	[1.79–2.82]	< 0.001	2.13	[1.56–2.91]	< 0.001	2.02	[1.49–2.73]	< 0.001
Total cholesterol (every 10 mg/dL)	0.96	[0.95–0.97]	< 0.001						
Log_creatinine	1.96	[1.59–2.41]	< 0.001						
eGFR (every 10 mL/min/1.73 m^2^)	0.86	[0.83–0.90]	< 0.001	0.90	[0.85–0.95]	< 0.001			
Log_urinary protein (g/gCr)	0.89	[0.72–1.11]	0.31						
Log_urinary protein (g/day)	0.81	[0.65–1.01]	0.061	0.78	[0.59–1.04]	0.093	0.81	[0.61–1.08]	0.146
urinary occult blood present^a^	2.07	[1.55–2.78]	< 0.001						
urinary RBC present^a^	2.65	[2.06–3.41]	< 0.001						
Hematuria present^a^	2.65	[2.06–3.42]	< 0.001	1.92	[1.36–2.69]	< 0.001	2.18	[1.56–3.04]	< 0.001

*FSGS* focal segmental glomerulosclerosis, *OR* odds ratio, *CI* confidence interval, *BP* blood pressure, *eGFR* estimated glomerular filtration rate, *RBC* red blood cell, *HPF* high powered field.

^a^Definition: Urinary occult blood present, (1+), (2+), (3+) on dipstick; Urinary RBC present, ≥ 5/HPF in urine sediment; Hematuria present, (1+), (2+), (3+) on dipstick and ≥ 5/HPF in sediment.

^b^Multivariable model 1: Adjusted for systolic blood pressure, albumin, eGFR, daily urinary protein (log-transformed), hematuria.

^c^Multivariable model 2: Adjusted for age, systolic blood pressure, albumin, daily urinary protein (log-transformed), hematuria.

## Discussion

The present study used the data from a Japanese nationwide kidney biopsy registry. This is a unique study focusing on the clinical manifestations of FSGS with a large sample size. Furthermore, this study is the first one to describe the clinical features of primary FSGS from two different viewpoints: age stratification and comparison between nephrotic FSGS and MCD.

In the J-RBR database, FSGS occupied 3.4–4.5% in total number of kidney biopsy. The Research Group on Progressive Renal Disease from the Ministry of Health, Labor and Welfare of Japan reported that the annual number of native kidney biopsy in Japan was estimated 18,000 to 21,000^20^. Therefore, approximately 800 patients per year were inferred to be diagnosed with FSGS by biopsy in our country. The prevalence of FSGS varied among races and countries^21^. An international survey revealed that FSGS accounted for 19.1% of primary glomerular disease in North America, 14.9% in Europe, 6.9% in Asia, and 15.8% in Latin America^22^. A part of this epidemiological difference could be explained by the presence of genetic variants in *apolipoprotein L1 (APOL1)* among people with sub-Saharan ancestry^23^. Additionally, several studies reported that the incidence of FSGS was increasing, especially in the US^24–26^. The present study shows that incidence of FSGS in Japan was lower than that in reports from other regions^21^ and it had not changed for the past decade.

To evaluate the population with FSGS, it is necessary to distinguish primary FSGS from secondary FSGS^27^. However, distinguishing secondary FSGS is challenging and the incidence of secondary FSGS remains unclear. D’Agati reported that 10–20% of FSGS patients are secondary ones^3^. In J-RBR, secondary cases constituted 16.2% of the total FSGS patients, and our results showed that the etiologies in these patients were different among the age groups; 10.8%, 17.4% and 16.0% among the pediatric, adult and elderly group, respectively. As the etiology, hypertension and obesity were the most common in total or among the adult and elderly group. Unilateral kidney or renal dysplasia, low birth weight and genetic disorders were leading causes of FSGS among the pediatric group (Supplementary Fig. S3).

Analysis 1 described the clinical characteristics of primary FSGS in three age groups. We showed that the proportion of NS was different in each age group. Previous studies reported that nephrotic-range proteinuria is more frequently seen in children than in adults in FSGS^8^. In our study, the pediatric group showed the highest prevalence of NS (73.5%). However, the urinary protein level was the lowest in this group. This discrepancy between clinical diagnosis and laboratory data could be explained by the effect of immunosuppressive treatment. Because of the quite high occupancy of MCD in NS of pediatric age, children with NS typically receive empiric treatment centering glucocorticoids and the indication of kidney biopsy are limited to the cases with refractory clinical course i.e., steroid-resistant or frequent relapse. Therefore, it is possible that most pediatric FSGS patients had already received immunosuppressive treatment by the time of biopsy. However, we cannot distinguish whether the data was obtained before or after initiation of immunosuppressive treatment, in the J-RBR data. The adult group showed the lowest prevalence of NS (41.2%) and the highest prevalence of chronic nephritic syndrome (54.3%). Whereas, the elderly group showed a higher prevalence of NS (65.7%) than the adult group. This suggested the differences in indications for renal biopsy among age groups. In Japan, a nationwide health examination program for all community residents, including urinalysis screening, has been conducted for over 40 years^28^. This regular screening enables early detection of urine abnormalities and early referral to a nephrologist. The suggested indication for renal biopsy in adult patients in Japan is ≥ 0.5 g/day of proteinuria or any amount of proteinuria with hematuria^29^. Many patients in the adult group may have undergone biopsy based on these suggested indications. Therefore, it is likely that the non-nephrotic cases with a substantial number of secondary FSGS were included to this age group. In contrast, it was also possible that the threshold of indications for renal biopsy in elderly patients might be higher i.e., clinicians attempted to perform biopsy for NS patients but not for the non-nephrotic patients in this age group. The clinical features of overall FSGS patients including secondary FSGS in the J-RBR and their age-group comparison were shown in Supplementary Table S4.

Analysis 2 compared the clinical features of nephrotic FSGS and MCD at biopsy. In this analysis, nephrotic FSGS was considered as primary FSGS because their laboratory data (nephrotic proteinuria with hypoalbuminemia) were consistent with NS^5,30^ and secondary cases with identifiable etiologies had been excluded. Even though classic articles suggested that hypertension, renal insufficiency and hematuria at the onset of disease are helpful to distinguish FSGS from MCD^1^, none have ever been statistically evaluated for their clinical significance in FSGS with a large sample size. We found that higher blood pressure, higher serum albumin, and incidence of hematuria at biopsy were associated with nephrotic FSGS in two multivariable models. FSGS was associated with older age and lower eGFR in univariate analysis. However, association between higher age and FSGS was not observed in multivariable Model 2, which suggested that there was confounding between age and eGFR. As described above, pediatric patients could represent biased population with the necessity of single or repeat biopsy for steroid-resistant or frequent relapse NS. We conducted sub-analysis only including the patients aged ≥ 18 years (nephrotic FSGS: n = 277, MCD: n = 1135) and obtained similar results as original analysis (Supplementary Tables S5, S6). These results suggest that lower eGFR but not higher age was an independent factor discriminating primary FSGS from MCD.

This study has several limitations. First, the J-RBR system did not collect detailed information regarding the etiology of FSGS. All the information was provided by the local coinvestigators. The J-RBR also lacked the information of the findings in electron microscopy and detailed genetic testing that could help discriminating primary and secondary FSGS. It is possible that primary FSGS may include some secondary cases and, therefore, the percentage of secondary FSGS may be underestimated in our study. Second, the J-RBR did not collect the information about total number of glomeruli. Therefore, it was possible that we included the patients without adequate number of glomeruli to distinguish FSGS from MCD in the present study. Third, this study was based on cross-sectional data. Although eGFR was lower in FSGS than in MCD, it was difficult to determine whether the impaired kidney function indicated chronic kidney disease or acute kidney injury. Fourth, as mentioned above, the J-RBR data do not include information on whether the data were obtained before or after the initiation of immunosuppressive treatment. Modification of the J-RBR system has been conducted to achieve a more accurate description of pathological diagnoses, including etiological information^31^. Another limitation of this study is the lack of information regarding pathological subgroups of FSGS: variants of the Columbia classification^27^. Previous studies reported that morphologic variants of FSGS demonstrate distinct features in their clinical presentation and prognosis^32–37^. A longitudinal investigation is currently underway based on J-RBR database including the additional information regarding Columbia classification.

In conclusion, nation-wide registry system revealed the characteristics and clinical features of FSGS in Japan. The results show that the incidence of FSGS in Japan is lower than that in other countries. When primary FSGS patients were divided into three age groups, the incidence of NS was lowest in the adult group (18–64 years), suggesting that the indications for renal biopsy might have been stricter among pediatric and elderly patients. When the analysis was focused on primary NS at the time of renal biopsy, FSGS showed distinct clinical features, such as younger age, higher blood pressure, higher serum albumin, lower eGFR, lower daily urinary protein level and presence of hematuria compared with MCD.

## Methods

### Overview of the J-RBR system

This cross-sectional study used the data from the J-RBR. The details of the J-RBR system were described in a previous publication^10^. As of June 2016, 143 nephrology centers participated in this registry, which included 30,949 patients. The J-RBR enters patient clinical information at biopsy. The J-RBR diagnosis consists of three components: (i) a clinical diagnosis, (ii) a histological diagnosis by pathogenesis, and (iii) a histological diagnosis by histopathology.

### Patients

Among the patients who registered to J-RBR between July 2007 and June 2016, cases whose histological diagnosis (histopathology) was “focal segmental glomerulosclerosis” were extracted for the evaluation of FSGS. Additionally, patients whose histological diagnosis (histopathology) was “minor glomerular abnormalities” were also extracted for the evaluation of MCD (Fig. 1).

### General demographics of FSGS in the J-RBR

Primary FSGS and secondary FSGS in the J-RBR database were included. From the patients with histological diagnosis (histopathology) of “focal segmental glomerulosclerosis” without multiple registration (n = 1409), the patients whose histological diagnosis (pathogenesis) was “primary glomerular disease” were defined as primary FSGS unless they had information of etiologies. The patients with identified information of their etiologies (i.e. obesity, hypertension, etc.) were defined as secondary FSGS. For example, when hypertension was considered to be a cause of FSGS, the case was classified into secondary FSGS. When hypertension was considered as just a concomitant condition, the case was classified into primary FSGS. FSGS lesions in other diseases (i.e. diabetic nephropathy, lupus nephritis, etc.) and renal graft were excluded. The details of selection for FSGS patients were described in Supplementary Fig. S1 and Supplementary Table S1.

The annual incidence of FSGS and proportion of primary and secondary FSGS and were described. Then, two main analyses were conducted as follows.

### Analysis 1: description of clinical features of primary FSGS in the three age groups

The cases with primary FSGS were divided into three age groups: pediatric group (< 18 years), adult group (18–64 years), and elderly group (≥ 65 years) (Fig. 1). Clinical parameters were compared among the age groups.

### Analysis 2: comparison between nephrotic FSGS and MCD

To compare the clinical characteristics of nephrotic FSGS with those of MCD, patients who fulfilled the following criteria were included in Analysis 2 (Fig. 1).

#### Nephrotic FSGS

(i) primary cases, (ii) clinical diagnosis of NS, and (iii) laboratory data consistent with NS, which was defined as urinary protein ≥ 40 mg/h/m^2^ or ≥ 2.0 g/gCr and serum albumin ≤ 2.5 g/dL for pediatric patients (age < 18) and urinary protein ≥ 3.5 g/day or ≥ 3.5 g/gCr and serum albumin ≤ 3.0 g/dL for adult and elderly patients (age ≥ 18). The body surface area (m^2^) of pediatric patients was calculated by the DuBois formula: height (cm)^0.725^ × body weight (kg)^0.425^ × 0.007184.

#### Nephrotic MCD

From the patients with histological diagnosis (histopathology) of “minor glomerular abnormalities”, following patients were included: (i) primary MCD, (ii) clinical diagnosis of NS, and (iii) laboratory data consistent with NS. Patients with multiple registrations, secondary MCD, other diseases with no obvious light microscopic glomerular findings (i.e., thin basement membrane disease, Class I lupus nephritis, etc.), and renal graft were excluded. The details of selection for MCD patients were described in Supplementary Fig. S2 and Supplementary Table S2.

Clinical parameters were compared between nephrotic FSGS and MCD patients.

### Data collection

Clinical data at biopsy such as patient characteristics (age, sex, height, body weight, blood pressure), comorbidities (concomitant hypertension and diabetes), urinary findings (urinalysis, daily proteinuria), and blood test findings (serum creatinine, total protein, albumin, total cholesterol) were extracted from the J-RBR database.

eGFR was calculated using equations based on serum creatinine (sCr) level for Japanese children (age < 18)^38^: eGFR [mL/min/1.73 m^2^] = 110.2 × reference-sCr/sCr (mg/dL) + 2.93, reference-sCr =  − 1.259 × Height (m)^5^ + 7.815 × Height (m)^4^ − 18.57 × Height (m)^3^ + 21.9 × Height (m)^2^ − 11.71 × Height (m) + 2.628 (if male), reference sCr =  − 4.536 × Height (m)^5^ + 27.16 × Height (m)^4^ − 63.47 × Height (m)^3^ + 72.43 × Height (m)^2^ − 40.06 × Height (m) + 8.778 (if female) and adults (age ≥ 18)^39^: eGFR [mL/min/1.73 m^2^] = 194 × sCr (mg/dL)^−1.094^ × Age^−0.287^ (if male), eGFR [mL/min/1.73 m^2^] = 194 × sCr (mg/dL)^−1.094^ × Age^−0.287^ × 0.739 (if female). Additional diagnostic information, including details of etiology were also extracted.

## Statistics

### Analysis 1: description of clinical features of primary FSGS in the three age groups

Clinical characteristics were expressed as median/interquartile range and frequency number/percentage. We used the Kruskal–Wallis test to compare continuous variables and the chi-squared test to compare the proportions of categorical variables among the age groups.

### Analysis 2: comparison between nephrotic FSGS and MCD

We used the Mann–Whitney U test to compare continuous variables and the chi-squared test to compare the proportions of categorical variables between nephrotic FSGS and MCD. Logistic regression analysis was used to evaluate the association between each variable and diagnosis of FSGS compared to MCD. Variables with P < 0.05 in univariate analysis and variables with clinical importance (age, eGFR, and urinary protein level) were included in the multivariable model, while we excluded variables with strong correlations (Pearson’s correlation coefficients > 0.40) or clinical relevance.

Statistical analyses were conducted using STATA IC version 14.0 (StataCorp LLC, College Station, TX, USA). The statistical significance level was set at P < 0.05.

### Ethics

Written informed consent was obtained from all participants and/or their legal guardians. All procedures performed in the present study were in accordance with the standards of the ethics committee of the Japanese Society of Nephrology (approval number: 40, J-RBR201604) and the ethics committee of Nagoya University (approval number: 2016-0492-2), and with the Helsinki Declaration of 1975 and its later amendments. J-RBR is registered in the UMIN Clinical Trial Registry (UMIN000000618).

## Supplementary Information

Supplementary Information.

## Data Availability

The datasets generated and analyzed during the current study are not publicly available because the consent obtained from the participants does not cover unlimited public sharing of the data but are available from the corresponding author on reasonable request.
