# Supplementary material for: Clinical manifestations of focal segmental glomerulosclerosis in Japan from the Japan Renal Biopsy Registry: age stratification and comparison with minimal change disease
Source: Sci Rep. 2021 Jan 28;11:2602. doi: 10.1038/s41598-020-80931-9 (PMC7844271; doi:10.1038/s41598-020-80931-9)
Supplement: Supplementary file 1 — Supplementary Information. [file 41598_2020_80931_MOESM1_ESM.pdf]

Supplementary Information

Clinical manifestations of focal segmental glomerulosclerosis in Japan from the Japan Renal Biopsy Registry: Age stratification and comparison with minimal change disease

Takaya Ozeki[1], Shoichi Maruyama[1]*, Toshiyuki Imasawa[2], Takehiko Kawaguchi[2], Hiroshi Kitamura[3], Moritoshi Kadomura[2], Ritsuko Katafuchi[4,5], Kazumasa Oka[6], Hitoshi Yokoyama[7], Hitoshi Sugiyama[8], Hiroshi Sato[9], on behalf of the Committee for Renal Biopsy and Disease Registry of the Japanese Society of Nephrology

[1] Department of Nephrology, Nagoya University Graduate School of Medicine, Nagoya, Japan
[2] Department of Nephrology, National Hospital Organization Chibahigashi National Hospital, Chiba, Japan
[3] Department of Pathology, National Hospital Organization Chibahigashi National Hospital, Chiba, Japan
[4] Department of Nephrology, Medical Corporation Houshikai Kano Hospital, Fukuoka, Japan
[5] Kidney Unit, National Hospital Organization Fukuoka Higashi Medical Center, Fukuoka, Japan
[6] Department of Pathology, Hyogo Prefectural Nishinomiya Hospital, Hyogo, Japan
[7] Department of Nephrology, Kanazawa Medical University School of Medicine, Ishikawa, Japan
[8] Department of Human Resource Development of Dialysis Therapy for Kidney Disease, Okayama University Graduate School of Medicine, Dentistry and Pharmaceutical Sciences, Okayama, Japan
[9] Department of Internal Medicine, Sendai Hospital of East Japan Railway Company, Sendai, Japan

*Corresponding author: Shoichi Maruyama
Department of Nephrology Graduate School of Medicine, Nagoya University, 65 Tsurumai-cho, Showa-ku, Nagoya 466-8550, Japan
Tel: +81-(0)52-744-2192, Fax: +81-(0)52-744-2209
E-mail: marus@med.nagoya-u.ac.jp

Supplementary Figure S1. Overview of the histologically diagnosed "focal segmental glomerulosclerosis" in the J-RBR

Supplementary Table S1. Details of the distribution of histopathologically diagnosed "focal segmental glomerulosclerosis" in the J-RBR

Supplementary Figure S2. Overview of the histologically diagnosed "minor glomerular abnormalities" in the J-RBR

Supplementary Table S2. Details of the distribution of histopathologically diagnosed "minor glomerular abnormalities" in the J-RBR

Supplementary Figure S3. Distribution and details of FSGS etiologies in the J-RBR

Supplementary Figure S4. Age-stratified distribution and proportion of the patients with FSGS and MCD in the J-RBR

Supplementary Table S3. Correlations between clinical continuous variables

Supplementary Table S4. Clinical features of FSGS in the J-RBR

Supplementary Table S5. Comparison between FSGS and MCD cases among adult and elderly patients (age≥18) with nephrotic syndrome

Supplementary Table S6. Associating factors with FSGS vs. MCD among adult and elderly patients (age≥18) with nephrotic syndrome

Supplementary Note. The investigators and institutions participating to the Japan Renal Biopsy Registry

Supplementary Figure S1. Overview of the histologically diagnosed “focal segmental glomerulosclerosis” in the J-RBR

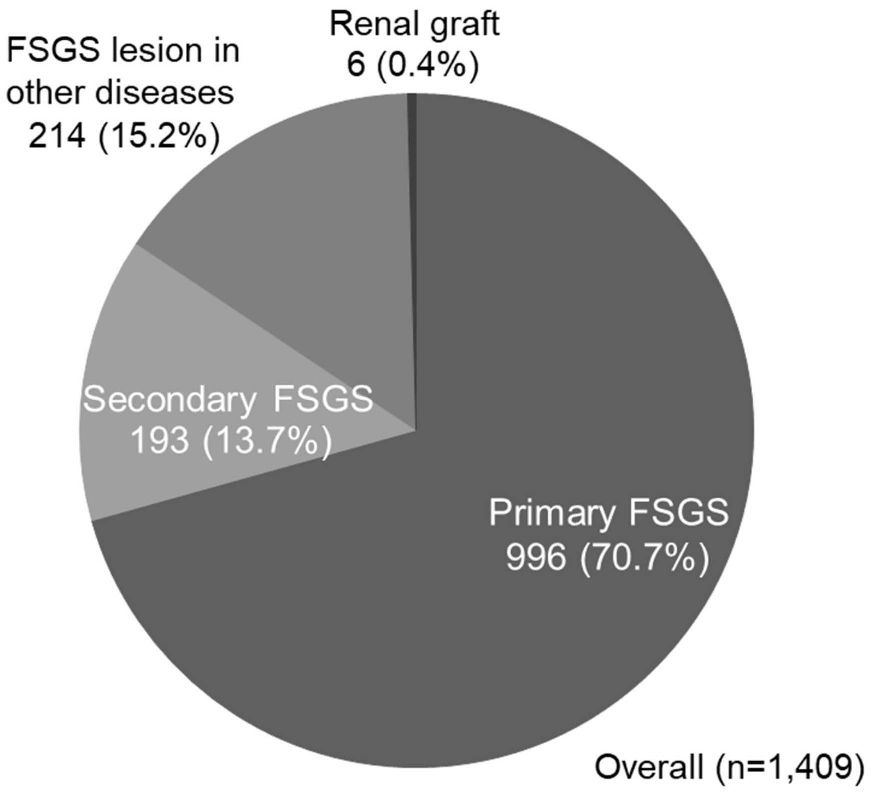

Registration in the J-RBR system has 3 diagnostic components: (i) clinical diagnosis, (ii) histological diagnosis by pathogenesis, and (iii) histological diagnosis by histopathology. The details of diagnostic system in the J-RBR should be referred to Sugiyama H et al., Clin Exp Nephrol 2011;15493-503.

From 30,949 patients who were registered to the J-RBR, 1,409 patients whose histological diagnosis by histopathology was “focal segmental glomerulosclerosis” were extracted for the analyses of FSGS. They included four types of patients as below.

Primary FSGS; Patients who were registered as “primary glomerular disease” in histological diagnosis (pathogenesis) without any information explaining the etiology of secondary cases.
Secondary FSGS; Patients who were registered with any information regarding the etiology of FSGS. Patients in this group could be identified only when the case has been registered with optional information about etiology.
FSGS lesion in other diseases; Patients with other type of systemic or glomerular diseases who had nonspecific segmental scarring, i.e. diabetic nephropathy, lupus nephritis and so on. Patients with any type of histological diagnosis (pathogenesis) except for “primary glomerular disease” were categorized to this group unless there were any information regarding secondary cases.
Renal graft: Patients whose clinical diagnosis was “renal transplantation” or whose histological diagnosis (pathogenesis) was “transplanted kidney”.

Abbreviations: J-RBR, Japan Renal Biopsy Registry; FSGS, focal segmental glomerulosclerosis

Supplementary Table S1. Details of the distribution of histopathologically diagnosed “focal segmental glomerulosclerosis” in the J-RBR

(A) Details of patient category

**Histological diagnosis by pathogenesis**	**n**	**Primary FSGS**	**Secondary FSGS**	**FSGS lesion in other diseases**	**Renal graft**
Primary glomerular disease*	1,024	996	28		
Hypertensive nephrosclerosis †	75		75		
Diabetic nephropathy	31			31	
Lupus nephritis	26			26	
Transplanted kidney ‡	6				6
Thin basement membrane disease	6			6	
Infection-related nephropathy	2		1	1	
Purpura nephritis	2			2	
Thrombotic microangiopathy	2			2	
Others	235		89	146	
Total	1,409	996	193	214	6

*Patients whose histological diagnosis (pathogenesis) was “primary glomerular disease” were categorized to “**Primary FSGS**” in main analyses unless they had any information about the etiology of FSGS.

Patients who were registered with any information regarding the etiology of FSGS were categorized to “**Secondary FSGS**” in main analyses.

†Patients whose histological diagnosis (pathogenesis) was “hypertensive nephrosclerosis” were categorized to “**Secondary FSGS**” as hypertension related FSGS in main analyses.

‡Patients whose histological diagnosis (pathogenesis) was “transplant kidney” were categorized to “**Renal graft**” and excluded from main analyses.

Patients who had any histological diagnosis (pathogenesis) except above were categorized to “**FSGS in other diseases**” and excluded from main analyses unless they had any information about the etiology of FSGS.

Abbreviations: J-RBR, Japan Renal Biopsy Registry; FSGS, focal segmental glomerulosclerosis

(B) Details of distribution on the secondary FSGS

**Histological diagnosis by pathogenesis**	**Secondary FSGS**	Hyper-tension	Obesity-related	Unilateral kidney or renal dysplasia	Low birth weight	Genetic	HIV infection	Cyanotic congenital heart disease	Drug-induced	Others or no detailed information
Primary glomerular disease	28		18	2	3	2				3
Hypertensive nephrosclerosis	75	75								
Infection-related nephropathy	1						1			
Others	89	2	54	8	4	4		1	1	15
Total	193	77	72	10	7	6	1	1	1	18

Patients in the "**Secondary FSGS**" could be identified when the case has been registered with optional information about etiology.

Abbreviations: FSGS, focal segmental glomerulosclerosis; HIV, human immunodeficiency virus

Supplementary Figure S2. Overview of the histologically diagnosed "minor glomerular abnormalities" in the J-RBR

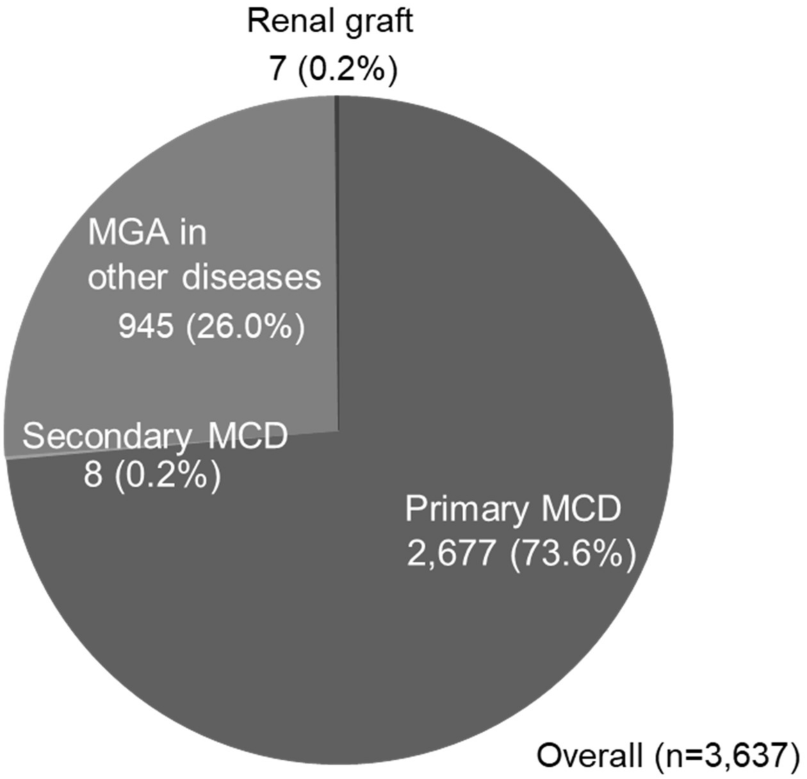

Registration in the J-RBR system has 3 diagnostic components: (i) clinical diagnosis, (ii) histological diagnosis by pathogenesis, and (iii) histological diagnosis by histopathology. The details of diagnostic system in the J-RBR should be referred to Sugiyama H et al., Clin Exp Nephrol 2011;15493-503.

From 30,949 patients who were registered to the J-RBR, 3,637 patients whose histological diagnosis by histopathology was "**minor glomerular abnormalities**" were extracted for the analyses of MCD. They included four types of patients as below.

Primary MCD; Patients who were registered as "primary glomerular disease" in histological diagnosis (pathogenesis) without any information about the etiology. In this category, the patients whose clinical diagnosis was NS and who fulfilled the laboratory criteria for NS, were included to **Analysis 2**.
Secondary MCD; Patients who were registered with any information regarding the etiology of MCD. Patients in this group could be identified when the case has been registered with optional information about etiology.
MGA in other diseases; Patients with other type of systemic or glomerular diseases who had no specific glomerular findings on light microscopy i.e. thin basement membrane disease, lupus nephritis (class I) and so on. Patients with any type of histological diagnosis (pathogenesis) except for "primary glomerular disease" were categorized to this group unless there were any information regarding secondary cases.
Renal graft: Patients whose clinical diagnosis was "renal transplantation" or whose histological diagnosis (pathogenesis) was "transplanted kidney".

Abbreviations: MCD, minimal change disease; J-RBR, Japan Renal Biopsy Registry; NS, nephrotic syndrome; MGA, minor glomerular abnormalities

Supplementary Table S2. Details of the distribution of histopathologically diagnosed "minor glomerular abnormalities" in the J-RBR

(A) Details of patient category

**Histological diagnosis by pathogenesis**	**n**	**Primary MCD**	**Secondary MCD**	**MGA in other diseases**	**Renal graft**
Primary glomerular disease*	2,679	2,677	2		
IgA nephropathy	187			187	
Thin basement membrane disease	118			118	
Hypertensive nephrosclerosis	42			42	
Alport syndrome	36			36	
Lupus nephritis	33			33	
Purpura nephritis	30			30	
Diabetic nephropathy	17			17	
MPO-ANCA positive nephritis	13			13	
Amyloid nephropathy	11			11	
Transplanted kidney †	7				7
PR3-ANCA positive nephritis	5			5	
Infection-related nephropathy	4			4	
Anti-GBM antibody nephritis	2			2	
Thrombotic microangiopathy	2			2	
Others	451		6	445	
Total	3,637	2,677	8	945	7

*Patients whose histological diagnosis (pathogenesis) was "primary glomerular disease" were categorized to "**Primary MCD**" in main analysis unless they had any information about the etiology of MCD.

Patients who were registered with any information regarding the etiology of MCD were categorized to "**Secondary MCD**" in main analysis.

Patients who had any histological diagnosis (pathogenesis) except for "primary glomerular disease" were categorized to "**MGA in other diseases**" and excluded from main analysis unless they had any information about the etiology of MCD.

†Patients whose histological diagnosis (pathogenesis) was "transplant kidney" were categorized to "**Renal graft**" and excluded from main analysis.

Abbreviations: J-RBR, Japan Renal Biopsy Registry; MCD, minimal change disease; MGA, minor glomerular abnormality

(B) Details of distribution on the secondary MCD

**Histological diagnosis by pathogenesis**	**Secondary MCD**	Malignancy	Hematological disorders	Drug-induced
Primary glomerular disease	2	3		
Others	6	1	2	3
Total	8	3	2	3

Patients in the "**Secondary MCD**" could be identified only when the case has been registered with optional information about etiology.

Abbreviations: MCD, minimal change disease

Supplementary Figure S3. Distribution and details of FSGS etiologies in the J-RBR

Overall (n=1,189)

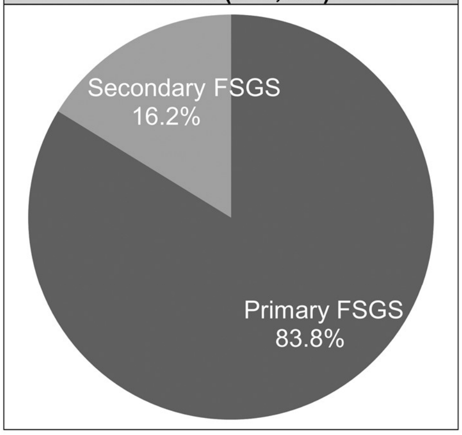

Etiologies of Secondary FSGS	n	(%)
**Overall (n=193/1,189)**		
Hypertension*	77	(39.9)
Obesity-related	72	(37.3)
Unilateral kidney or renal dysplasia	10	(5.2)
Low birth weight	7	(3.6)
Genetic†	6	(3.1)
HIV infection	1	(0.5)
Cyanotic congenital heart disease	1	(0.5)
Drug-induced‡	1	(0.5)
Others or no detailed information	18	(9.3)

Pediatric group (n=148)

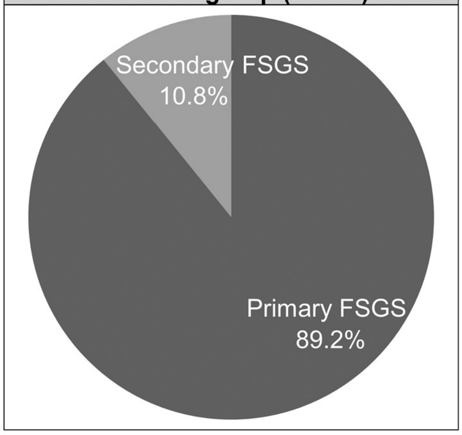

Etiologies of Secondary FSGS	n	(%)
**Pediatric group (n=16/148)**		
Unilateral kidney or renal dysplasia	5	(31.3)
Low birth weight	3	(18.8)
Genetic	3	(18.8)
Obesity-related	2	(12.5)
Hypertension	2	(12.5)
Cyanotic congenital heart disease	1	(6.3)

Adult group (n=753)

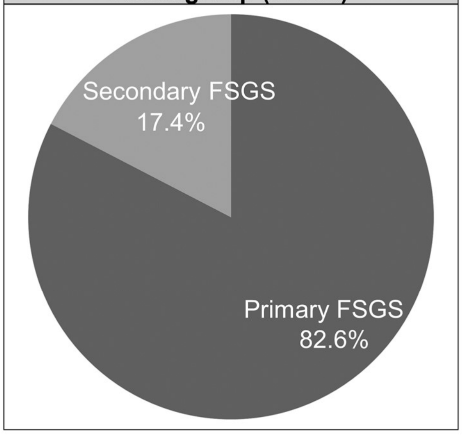

Etiologies of Secondary FSGS	n	(%)
**Adult group (n=131/753)**		
Obesity-related	63	(48.1)
Hypertension	47	(35.9)
Unilateral kidney or renal dysplasia	5	(3.8)
Low birth weight	4	(3.1)
Genetic	3	(2.3)
HIV infection	1	(0.8)
Others or no detailed information	8	(6.1)

Elderly group (n=288)

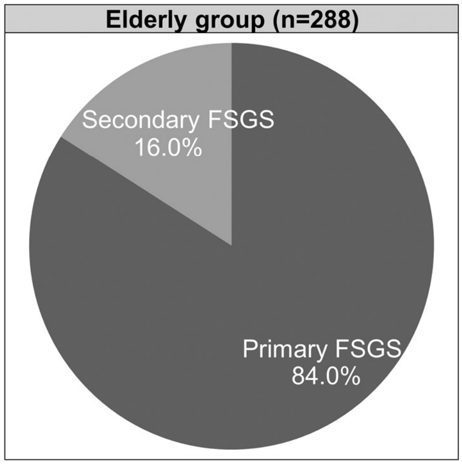

Etiologies of Secondary FSGS	n	(%)
**Elderly group (n=46/288)**		
Hypertension	28	(60.9)
Obesity-related	7	(15.2)
Drug-induced	1	(2.2)
Others or no detailed information	10	(21.7)

Information about etiology of FSGS was registered in 193 (16.2%) cases. The details of etiologies were shown by overall patients and three age groups: pediatric group (<18 years), adult group (18-64 years), and elderly group (≥65 years).
*Hypertension: cases whose histological diagnosis (pathogenesis) was "hypertensive nephrosclerosis"
†Genetic: no recorded information about mutations
‡Drug-induced: sunitinib

Abbreviations: FSGS, focal segmental glomerulosclerosis; J-RBR, Japan Renal Biopsy Registry; HIV, human immunodeficiency virus

Supplementary Figure S4. Age-stratified distribution and proportion of the patients with FSGS and MCD in the J-RBR

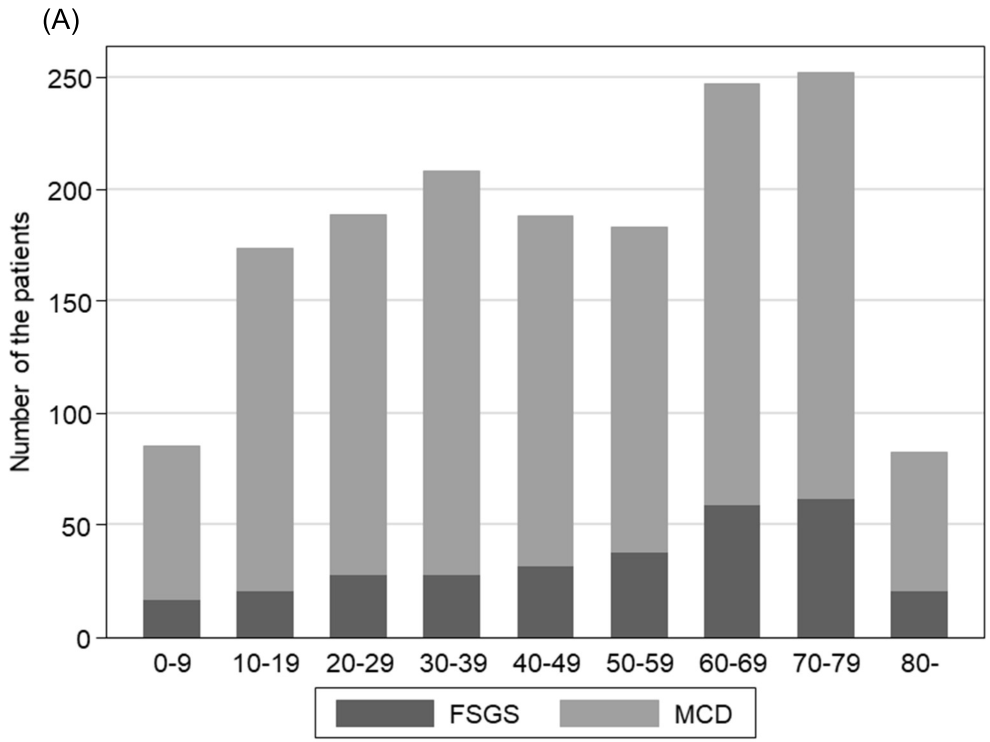

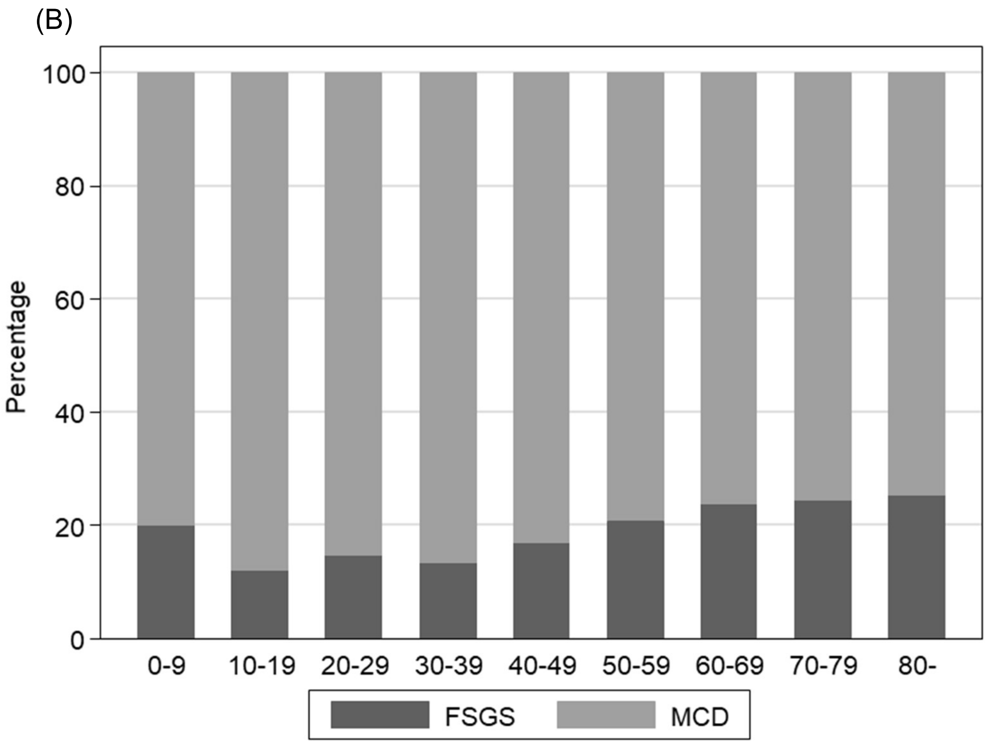

(A) Number of the patients with FSGS and MCD by age (B) Percentage of the patients with FSGS and MCD by age in the J-RBR.

Abbreviations: FSGS, focal segmental glomerulosclerosis; MCD, minimal change disease; J-RBR, Japan Renal Biopsy Registry

Supplementary Table S3. Correlations between clinical continuous variables

	Age	BMI	sBP	TP	Albumin	T-Cho	Log_Cre	eGFR	Log_UP (g/day)	Log_UP (g/gCr)
Age	**1.00**									
BMI	0.21	**1.00**								
sBP	0.36	0.27	**1.00**							
TP	0.16	-0.04	0.08	**1.00**						
Albumin	0.12	-0.08	0.20	0.40	**1.00**					
T-Cho	-0.28	0.02	-0.22	-0.26	**-0.44**	**1.00**				
Log_Cre	**0.44**	0.29	0.35	-0.04	-0.08	-0.17	**1.00**			
eGFR	**-0.61**	-0.26	-0.34	-0.01	0.08	0.14	**-0.86**	**1.00**		
Log_UP (g/day)	-0.15	0.15	0.01	-0.21	-0.23	0.14	0.15	-0.09	**1.00**	
Log_UP (g/gCr)	0.02	0.02	-0.01	-0.04	-0.21	0.08	0.06	-0.14	0.35	**1.00**

To avoid inclusion of variables with strong correlations in multivariate logistic regression, we evaluated correlations between continuous variables. Numbers in cells indicate Pearson's correlation coefficients. Correlation coefficients >0.40 or <-0.40 are shown in **bold font**.

Abbreviations: BMI, Body Mass Index; sBP, systolic blood pressure; TP, total protein, T-Cho, total cholesterol; Cre, creatinine; eGFR, estimated glomerular filtration rate; UP, urinary protein

Supplementary Table S4. Clinical features of FSGS in the J-RBR

	obs	Overall (n=1,189)		Age groups: Pediatric group (n=148)		Adult group (n=753)		Elderly group (n=288)		P-value
**Patient characteristics**										
Age	1,189	48	[29-64]	11	[5-15]	44	[32-55]	72	[68-77]	<0.001
Sex (male)	1,189	724	(60.9)	93	(62.8)	446	(59.2)	185	(64.2)	0.29
Number of biopsies										
First	1,189	637	(53.6)	59	(39.9)	404	(53.7)	174	(60.4)	<0.001
Second		73	(6.1)	25	(16.9)	39	(5.2)	9	(3.1)	
≥3 times		28	(2.4)	11	(7.4)	15	(2.0)	2	(0.7)	
Unknown		451	(37.9)	53	(35.8)	295	(39.2)	103	(35.8)	
Clinical diagnosis										
Nephrotic syndrome	1,189	535	(45.0)	97	(65.5)	264	(35.1)	174	(60.4)	<0.001
Chronic nephritic syndrome		577	(48.5)	41	(27.7)	436	(57.9)	100	(34.7)	
Others		77	(6.5)	10	(6.8)	53	(7.0)	14	(4.9)	
Secondary FSGS*	1,189	193	(16.2)	16	(10.8)	131	(17.4)	46	(16.0)	0.138
Body Mass Index	1,173	23.5	[20.5-27.0]	18.5	[16.2-21.9]	24.2	[21.0-28.1]	23.7	[21.6-26.0]	<0.001
Systolic blood pressure	1,004	130	[118-142]	116	[105-122]	129	[120-141]	138	[126-150]	<0.001
Diastolic blood pressure	1,004	78	[69-87]	70	[61-78]	80	[70-89]	77	[70-86]	<0.001
Antihypertensive drugs	984	526	(53.5)	35	(33.0)	316	(49.6)	175	(72.6)	<0.001
Diabetes mellitus	885	131	(14.8)	5	(5.3)	79	(13.9)	47	(21.8)	0.005
**Laboratory data**										
Total protein, g/dL	1,181	6.4	[5.2-7.1]	6.2	[5.2-7.0]	6.6	[5.3-7.1]	5.8	[4.9-6.8]	<0.001
Albumin, g/dL	1,178	3.6	[2.4-4.1]	3.8	[2.6-4.3]	3.8	[2.7-4.2]	2.8	[2.1-3.7]	<0.001
Total cholesterol, mg/dL	1,165	228	[190-302]	228	[172-369]	228	[193-291]	233	[191-314]	0.74
HbA1c (NGSP), %	742	5.7	[5.4-6.0]	5.5	[5.3-5.8]	5.7	[5.4-6.0]	5.8	[5.5-6.1]	0.009
Creatinine, mg/dL	1,187	0.98	[0.70-1.34]	0.49	[0.33-0.72]	0.95	[0.74-1.28]	1.26	[0.93-1.69]	<0.001

eGFR, mL/min/1.73 m^2	1,180	60	[40-83]	102	[80-131]	63	[46-82]	40	[29-57]	<0.001
Urinary protein, g/gCr	837	2.48	[0.95-6.04]	1.28	[0.16-5.18]	2.07	[0.88-4.70]	5.11	[2.33-9.16]	<0.001
Urinary protein, g/day	842	1.90	[0.73-4.90]	1.16	[0.20-5.47]	1.67	[0.70-4.24]	3.14	[1.10-5.90]	<0.001
Consistent with NS †	1,189	339	(28.5)	32	(21.6)	177	(23.5)	130	(45.1)	<0.001
Urinary occult blood										
(-)		420	(35.3)	84	(56.8)	263	(34.9)	73	(25.4)	
(±)		176	(14.8)	15	(10.1)	117	(15.5)	44	(15.3)	
(1+)	1,189	197	(16.6)	11	(7.4)	119	(15.8)	67	(23.3)	<0.001
(2+)		227	(19.1)	20	(13.5)	146	(19.4)	61	(21.2)	
(3+)		169	(14.2)	18	(12.2)	108	(14.3)	43	(14.9)	
Urinary occult blood present ‡	1,189	593	(49.9)	49	(33.1)	373	(49.5)	171	(59.4)	<0.001
Urinary RBC, /HFP										
(-)		197	(16.6)	39	(26.4)	118	(15.7)	40	(13.9)	
<5		545	(45.8)	56	(37.8)	351	(46.6)	138	(47.9)	
5-10	1,189	186	(15.6)	19	(12.8)	116	(15.4)	51	(17.7)	0.017
10-30		151	(12.7)	17	(11.5)	94	(12.5)	40	(13.9)	
many		110	(9.3)	17	(11.5)	74	(9.8)	19	(6.6)	
Urinary RBC present §	1,189	447	(37.6)	53	(35.8)	284	(37.7)	110	(38.2)	0.88
Hematuria present ‖	1,189	408	(34.3)	42	(28.4)	263	(34.9)	103	(35.8)	0.26

Data are presented as median [interquartile range] for continuous variables and count (percentage) for categorical variables.

* Details of secondary FSGS are summarized in Figure 3.

† Laboratory criteria for nephrotic syndrome, for pediatric patients (age <18): urinary protein ≥40 mg/hour/m^2 or ≥2.0 g/gCr and serum albumin ≤2.5 g/dL; for adult and elderly patients (age ≥18): urinary protein ≥3.5 g/day or ≥3.5 g/gCr and serum albumin ≤3.0 g/dL.

‡ Urinary occult blood present, (1+), (2+), (3+) on dipstick.

§ Urinary RBC present, ≥5/HPF in urine sediment.

‖ Hematuria present, (1+), (2+), (3+) on dipstick and ≥5/HPF in sediment.

Abbreviations: J-RBR, Japan Renal Biopsy Registry; obs, number of observations; eGFR, estimated glomerular filtration rate; NGSP, National Glycohemoglobin Standardization Program; NS, nephrotic syndrome; RBC, red blood cell; HPF, high powered field

Supplementary Table S5. Comparison between FSGS and MCD cases among adult and elderly patients (age≥18) with nephrotic syndrome

	Nephrotic FSGS age≥18 (n=277)			MCD age≥18 (n=1,135)			P-value
	obs	Median, n	[IQR], (%)	obs	Median, n	[IQR], (%)	
**Patient characteristics**							
Age	277	60	[40-72]	1,135	50	[33-68]	<0.001
Sex (male)	277	168	(60.7)	1,135	648	(57.1)	0.28
Body Mass Index	277	23.3	[20.9-26.4]	1,128	23.8	[21.4-26.7]	0.25
Systolic BP	246	138	[124-150]	996	124	[113-136]	<0.001
Diastolic BP	246	80	[70-90]	996	76	[68-84]	<0.001
Antihypertensive drugs	223	129	(57.9)	964	303	(31.4)	<0.001
Diabetes mellitus	198	32	(16.2)	873	131	(15.0)	0.68
**Laboratory data**							
Total protein, g/dL	275	4.7	[4.2-5.3]	1,131	4.5	[4.1-5.0]	<0.001
Albumin, g/dL	277	1.9	[1.5-2.4]	1,135	1.7	[1.3-2.1]	<0.001
Total cholesterol, mg/dL	273	331	[268-418]	1,130	406	[320-494]	<0.001
HbA1c (NGSP), %	202	5.6	[5.3-6.0]	845	5.6	[5.4-6.0]	0.159
Creatinine, mg/dL	275	1.10	[0.80-1.70]	1,134	0.89	[0.69-1.16]	<0.001
eGFR, mL/min/1.73 m^2	275	51.1	[30.3-68.9]	1,134	67.8	[48.0-84.6]	<0.001
Urinary protein, g/gCr	222	8.08	[5.52-10.79]	916	8.43	[5.60-11.57]	0.25
Urinary protein, g/day	216	6.28	[4.61-8.67]	902	7.02	[4.80-10.20]	0.006
Urinary occult blood							
(-)		24	(8.7)		194	(17.0)	
(±)		35	(12.6)		193	(17.1)	
(1+)	277	77	(27.8)	1,135	241	(21.2)	<0.001
(2+)		95	(34.3)		376	(33.1)	
(3+)		46	(16.6)		131	(11.5)	
Urinary occult blood present*	277	218	(78.7)	1,135	748	(65.9)	<0.001
Urinary RBC, /HPF							
(-)		20	(7.2)		153	(13.5)	
<5		112	(40.4)		609	(53.7)	
5-10	277	74	(26.7)	1,135	193	(17.0)	<0.001
10-30		46	(16.6)		131	(11.5)	
many		25	(9.0)		49	(4.3)	
Urinary RBC present*	277	145	(52.4)	1,135	373	(32.9)	<0.001
Hematuria present*	277	142	(51.3)	1,135	351	(30.9)	<0.001

*Definition: Urinary occult blood present, (1+), (2+), (3+) on dipstick; Urinary RBC present, ≥5/HPF in urine sediment; Hematuria present, (1+), (2+), (3+) on dipstick and ≥5/HPF in sediment.

Abbreviations: FSGS, focal segmental glomerulosclerosis; MCD, minimal change disease; BP, blood pressure; eGFR, estimated glomerular filtration rate; RBC, red blood cell; HPF, high powered field

Supplementary Table S6. Associating factors with FSGS vs. MCD among adult and elderly patients (age≥18) with nephrotic syndrome

Variables	Univariate			Multivariable					
				Model 1†			Model 2‡		
	OR	[95%CI]	P-value	OR	[95%CI]	P-value	OR	[95%CI]	P-value
Age (every10 years)	1.16	[1.08–1.25]	<0.001				1.00	[0.91–1.10]	0.94
Sex (Male)	1.16	[0.89–1.51]	0.28						
Body Mass Index	1.00	[0.97–1.03]	0.95						
Systolic BP (every10 mmHg)	1.38	[1.28–1.49]	<0.001	1.24	[1.14–1.37]	<0.001	1.31	[1.19–1.44]	<0.001
Anti-hypertensive drugs	2.99	[2.22–4.04]	<0.001						
Diabetes mellitus	1.09	[0.72–1.67]	0.68						
HbA1c (NGSP)	0.82	[0.64–1.05]	0.111						
Total protein	1.47	[1.22–1.76]	<0.001						
Albumin	2.29	[1.81–2.91]	<0.001	2.17	[1.58–3.00]	<0.001	2.00	[1.46–2.72]	<0.001
Total cholesterol (every 10 mg/dL)	0.95	[0.94–0.96]	<0.001						
Log_creatinine	2.23	[1.77–2.81]	<0.001						
eGFR (every 10 mL/min/1.73 m^2)	0.82	[0.78–0.87]	<0.001	0.87	[0.81–0.93]	<0.001			
Log_urinary protein (g/gCr)	0.88	[0.68–1.13]	0.32						
Log_urinary protein (g/day)	0.77	[0.61–0.98]	0.029	0.78	[0.58-1.05]	0.099	0.81	[0.60-1.10]	0.180
urinary occult blood present*	1.91	[1.40–2.61]	<0.001						
urinary RBC present*	2.24	[1.72–2.93]	<0.001						
Hematuria present*	2.35	[1.80–3.07]	<0.001	1.74	[1.22–2.46]	0.002	2.02	[1.46–2.72]	<0.001

* Definition: Urinary occult blood present, (1+), (2+), (3+) on dipstick; Urinary RBC present, ≥5/HPF in urine sediment; Hematuria present, (1+), (2+), (3+) on dipstick and ≥5/HPF in sediment.

† Multivariable model 1: Adjusted for systolic blood pressure, albumin, eGFR, daily urinary protein (log-transformed), hematuria.

‡ Multivariable model 2: Adjusted for age, systolic blood pressure, albumin, daily urinary protein (log-transformed), hematuria.

Abbreviations: FSGS, focal segmental glomerulosclerosis; OR, odds ratio; CI, confidence interval; BP, blood pressure; eGFR, estimated glomerular filtration rate; RBC, red blood cell; HPF, high powered field

Supplementary Note. The investigators and institutions participating to the Japan Renal Biopsy Registry

The following investigators and initial institutions have participated in the development of the J-RBR since 2007: Hirofumi Makino and Hitoshi Sugiyama (Okayama University), Takashi Taguchi (Nagasaki University), Hitoshi Yokoyama (Kanazawa Medical University), Hiroshi Sato (Tohoku University; present institution: JR Sendai Hospital), Takao Saito (Fukuoka University; present institution: Sanko Clinic), Yoshie Sasatomi (Fukuoka University; present institution: Kanenokuma Hospital), Yukimasa Kohda (Kumamoto University; present institution: Hikarinomori Clinic), Shinichi Nishi (Niigata University; present institution: Kobe University), Kazuhiko Tsuruya (Kyushu University; present institution: Nara Medical University), Yutaka Kiyohara (Kyushu University; present institution: Hisayama Research Institute for Lifestyle Diseases), Hideyasu Kiyomoto (Kagawa University; present institution: Tohoku Medical Megabank Organization, Tohoku University), Hiroyuki Iida (Toyama Prefectural Central Hospital; present institution: Toyama Prefectural Rehabilitation Hospital), Tamaki Sasaki (Kawasaki Medical School), Makoto Higuchi (Shinshu University), Motoshi Hattori (Tokyo Women's Medical University), Kazumasa Oka (Osaka Kaisei Hospital; present institution: Hyogo Prefectural Nishinomiya Hospital), Shoji Kagami (The University of Tokushima Graduate School), Michio Nagata (University of Tsukuba), Tetsuya Kawamura (The Jikei University School of Medicine), Masataka Honda (Tokyo Metropolitan Children's Medical Center), Yuichiro Fukasawa (KKR Sapporo Medical Center; present institution: Sapporo City General Hospital), Atsushi Fukatsu (Kyoto University Graduate School of Medicine; present institution: Fukatsu Medical Clinic), Kunio Morozumi (Japanese Red Cross Nagoya Daini Hospital; present institution: Masuko Memorial Hospital), Norishige Yoshikawa (Wakayama Medical University), Yukio Yuzawa (Fujita Health University), Seiichi Matsuo (Nagoya University) and Kensuke Joh (Chiba-East National Hospital; present institution: The Jikei University School of Medicine).

Hokkaido District

・Asahikawa Medical University Hospital (Division of Cardiology, Nephrology, Pulmonology and Neurology, Department of Internal Medicine), Naoyuki Hasebe, Naoki Nakagawa

・National Hospital Organization Hokkaido Medical Center (Department of Nephrology), Sekiya Shibazaki, Tomotsune Miyamoto, Masanori Ito

・Hokkaido University Graduate School of Medicine (Department of Rheumatology, Endocrinology and Nephrology, Faculty of Medicine and Graduate School of Medicine, Hokkaido University), Saori Nishio, Daigo Nakazawa

・Hokkaido University Graduate School of Medicine (Department of Pediatrics), Takayuki Okamoto, Yasuyuki Sato

・KKR Sapporo Medical Center (Department of Pathology), Akira Suzuki

・Sapporo Medical University (Department of Cardiovascular, Renal and Metabolic Medicine), Norihito Moniwa, Marenao Tanaka

・Sapporo City General Hospital, Yuichiro Fukasawa

・Teine Keijinkai Hospital (Department of Nephrology), Hideki Takizawa

Tohoku District

・Iwate Prefectural Central Hospital (Department of Nephrology and Rheumatology), Jun Soma, Izaya Nakaya

・Fukushima Medical University (Department of Nephrology and Hypertension), Junichiro James Kazama, Kennichi Tanaka, Mizuko Tanaka

・Japan Community Health care Organization Sendai Hospital (Department of Nephrology), Toshinobu Sato, Satoru Sanada, Hideaki Hashimoto

・Tohoku University Hospital and affiliated hospitals (Internal Medicine), Mariko Miyazaki, Tasuku Nagasawa, Koji

Okamoto

・Yamagata University School of Medicine (Department of Cardiology, Pulmonology, and Nephrology), Tsuneo Konta, Kazunobu Ichikawa

・Yamagata University School of Medicine (Department of Pediatrics), Daisuke Ogino

Kanto District

・National Hospital Organization Chibahigashi National Hospital (Department of Pathology), Hiroshi Kitamura, (Department of Nephrology), Toshiyuki Imasawa, (Department of Pediatrics), Chieko Matsumura, (Department of Surgery), Naotake Akutsu

・National Hospital Organization Chiba-East Hospital (Department of Urology), Koichi Kamura (*) *present address, Harunclinic Sakura

・Dokkyo Medical University Saitama Medical Center (Department of Nephrology), Tetsuro Takeda

・Dokkyo Medical University (Department of Nephrology and Hypertension), Toshihiko Ishimitsu

・Gunma University Graduate School of Medicine (Department of Nephrology and Rheumatology), Keiju Hiromura, Yoriaki Kaneko, Hidekazu Ikeuchi, Toru Sakairi

・Jichi Medical University (Division of Nephrology), Daisuke Nagata, Osamu Saito, Tetsu Akimoto, Akito Maeshima

・The Jikei University School of Medicine (Division of Nephrology and Hypertension) Takashi Yokoo, Nobuo Tsuboi, Kentaro Koike

・The Jikei University School of Medicine, Katsushika Medical Center (Division of Nephrology and Hypertension), Yudo Tanno, Shinya Yokote

・The Jikei University School of Medicine, Daisan Hospital (Division of Nephrology and Hypertension), Yoichi Miyazaki, Hiroyuki Ueda, Masahiro Okabe, Mai Tanaka

・The Jikei University Kashiwa Hospital (Division of Nephrology and Hypertension), Masato Ikeda, Akihiro Shimizu

・Juntendo University Faculty of Medicine (Department of Nephrology), Yusuke Suzuki, Tomohito Goda, Masao Kihara, Miyuki Takagi

・Kawaguchi Municipal Medical Center (Division of Nephrology), Masahiro Ishikawa

・Kyorin University School of Medicine (Department of Nephrology and Rheumatology), Shinya Kaname, Miho Karube, Kazuhito Fukuoka, Takahisa Kawakami

・Mito Saiseikai General Hospital (Division of Nephrology), Itaru Ebihara, Chihiro Sato

・Nippon Medical School (Division of Nephrology, Department of Internal Medicine), Yukinao Sakai, Akio Hirama, Akiko Mii

・Nihon University School of Medicine (Division of Nephrology, Hypertension and Endocrinology), Yoshinobu Fuke, Masanori Abe

・Saitama Medical University, Faculty of Medicine (Department of Nephrology), Hirokazu Okada, Tsutomu Inoue

・Saitama Medical University, Saitama Medical Center (Department of Nephrology and Hypertension), Takatsugu Iwashita, Yuta Kogure, Koichi Kanozawa, Hajime Hasegawa

・Saiyu Soka Hospital（Department of Internal Medicine), Masamitsu Ubukata

・Showa University School of Medicine (Division of Nephrology, Department of Medicine), Takanori Shibata

・Showa University Fujigaoka Hospital (Division of Nephrology, Department of Medicine), Yoshihiko Inoue

・St. Marianna University School of Medicine (Division of Nephrology and Hypertension, Department of Internal Medicine), Tomo Suzuki, Daisuke Ichikawa, Sayuri Shirai, Yugo Shibagaki

・Tokai University School of Medicine (Division of Nephrology, Endocrinology and Metabolism), Takehiko Wada, Masafumi

Fukagawa

・Teikyo University School of Medicine (Department of Internal Medicine), Yoshihide Fujigaki

・Teikyo University School of Medicine (Department of Urology), Shigeo Horie(*), Satoru Muto(*) *present address, Juntendo University School of Medicine (Department of Urology)

・Tokyo Medical University Ibaraki Medical Center (Department of Nephrology), Masaki Kobayashi, Kouichi Hirayama, Homare Shimohata

・Tokyo Metropolitan Children's Medical Center (Department of Nephrology), Riku Hamada (Department of General Pediatrics), Hiroshi Hataya

・Tokyo Women's Medical University (Department of Pediatric Nephrology), Motoshi Hattori, Kenichiro Miura, Kiyonobu Ishizuka, Naoto Kaneko

・Tokyo Women's Medical University (Department of Nephrology), Kosaku Nitta, Keiko Uchida, Takahito Moriyama

・Toranomon Hospital, Nephrology Center, Yoshifumi Ubara, Tatsuya Suwabe, Junichi Hoshino, Noriko Hayami

・The University of Tokyo (Department of Nephrology and Endocrinology), Masaomi Nangaku, Tetsuhiro Tanaka, Yoshifumi Hamasaki, Kenjiro Honda

・The University of Tokyo(Department of Pediatrics), Yutaka Harita, Shoichiro Kanda, Yuko Kajiho

・University of Tsukuba (Department of Nephrology), Kunihiro Yamagata, Joichi Usui, Tetsuya Kawamura

・Yokohama City University Graduate School of Medicine (Department of Medical Science and Cardiorenal Medicine), Kouichi Tamura, Hiromichi Wakui, Tomohiko Kanaoka, Ryu Kobayashi

・Yokohama City University Medical Center, Nobuhito Hirawa, Sanae Saka, Akira Fujiwara

Koushinetsu District

・Niigata University Graduate School of Medical and Dental Sciences (Division of Clinical Nephrology and Rheumatology), Ichiei Narita, Shin Goto, Yumi Itoh, Naofumi Imai

・Shinshu University School of Medicine (Department of Nephrology), Yuji Kamijo, Koji Hashimoto, Akinori Yamaguchi, Sonoda Kosuke

・University of Yamanashi Hospital (Third Department of Internal Medicine), Kazuya Takahashi, Fumihiko Furuya

Hokuriku District

・National Hospital Organization Kanazawa Medical Center (Department of Nephrology and Rheumatology), Kiyoki Kitagawa

・Kanazawa Medical University School of Medicine (Department of Nephrology), Hitoshi Yokoyama, Keiji Fujimoto, Norifumi Hayashi

・Kanazawa Medical University (Department of Diabetology & Endocrinology), Daisuke Koya, Munehiro Kitada, Yuka Kuroshima

・Kanazawa University Hospital (Division of Nephrology), Takashi Wada, Kengo Furuichi, Miho Shimizu, Norihiko Sakai

・Komatsu Sophia Hospital, Yasuhiro Katou, Yuta Yamamura

・Koshino Internal Medicine Clinic, Yoshitaka Koshino

・Pubulic Central Hospital of Matto-Ishikawa, Chikako Takaeda

・Sugita Genpaku Memorial Obama Municipal Hospital, Haruyoshi Yoshida, Takayasu Horiguchi

・Toyama Prefectural Central Hospital (Departent of Internal Medicine), Yasuyuki Shinozaki, Masahiko Kawabata

・Toyama City Hospital (Department of Internal Medicine), Satoshi Ota, Yoh-ichi Ishida

・University of Fukui, Faculty of Medical Sciences (Department of Nephrology), Masayuki Iwano, Naoki Takahashi, Kenji

Kasuno, Daisuke Mikami

・University of Toyama (Second Department of Internal Medicine), Hidenori Yamazaki

Tokai District

・Aichi Children's Health and Medical Center (Department of Pediatric Nephrology), Naoya Fujita, Satoshi Hibino, Kazuki Tanaka

・Aichi Medical University School of Medicine (Division of Nephrology and Rheumatology), Yasuhiko Ito, Takuhito Nagai, Takayuki Katsuno, Hironobu Nobata

・Chuno Kosei Hospital, Shogo Kimura, Yuka Soga

・Fujinomiya City General Hospital, Masanori Sakakima

・Fujita Health University School of Medicine (Department of Nephrology), Yukio Yuzawa, Naotake Tsuboi, Hiroki Hayashi, Kazuo Takahashi

・Hamamatsu University School of Medicine, University Hospital (Internal Medicine1, Division of Nephrology), Hideo Yasuda, Naro Ohashi, Taichi Sato

・Japanese Red Cross Nagoya Daini Hospital (Kidney Center), Asami Takeda, Yasuhiro Otsuka

・Nagoya City East Medical Center, Minamo Ono, Tatsuya Tomonari

・Nagoya City University Graduate School of Medical Sciences (Department of Cardio-Renal Medicine and Hypertension), Michio Fukuda, Masashi Mizuno, Taisei Suzuki, Satoru Kominato

・Nagoya Kyoritsu Hospital (Department of Internal Medicine), Hirotake Kasuga

・Nagoya University Graduate School of Medicine (Department of Nephrology), Shoichi Maruyama, Yoshinari Yasuda, Tomoki Kosugi, Takuji Ishimoto

・Shizuoka General Hospital (Department of Nephrology), Noriko Mori, Satoshi Tanaka

・Mie University Graduate School of Medicine (Department of Cardiology and Nephrology), Tomohiro Murata, Mika Fujimoto, Kan Katayama

・Japan Community Health care Organization Yokkaichi Hazu Medeical Center (Division of Nephrology and Blood Purification), Yasuhide Mizutani, Hitoshi Kodera, Masato Miyake

Kinki District

・Hyogo Prefectural Nisihinomiya Hospital (Department of Pathology), Kazumasa Oka

・Ikeda City Hospital (Department of Nephrology), Nobuyuki Kajiwara

・Kitano Hospital, Tazuke Kofukai Medical Research Institute (Department of Nephrology and Dialysis), Tatsuo Tsukamoto, Tomomi Endo, Eri Muso

・Kobe University Graduate School of Medicine (Division of Nephrology and Kidney Center), Shinichi Nishi, Shunsuke Goto

・Kobe University Graduate School of Medicine (Department of Pediatrics), Kazumoto Iijima, Kandai Nozu, Tomohiko Yamamura

・Japan Community Health care Organization Kobe Central Hospital, Yoko Adachi, Takaaki Nishihara, Michitsugu Kamezaki

・National Hospital Organization Kyoto Medical Center (Division of Nephrology), Koichi Seta, Kensei Yahata

・Kyoto Prefectural University of Medicine Graduate School of Medical Science (Department of Nephrology), Keiichi Tamagaki, Tetsuro Kusaba, Yayoi Shiotsu

・Kyoto University Graduate School of Medicine (Department of Nephrology), Motoko Yanagita, Hideki Yokoi, Kaoru Sakai, Akira Ishii

・Nara Medical University (Department of Nephrology), Kazuhiko Tsuruya, Kenichi Samejima
・National Cerebral and Cardiovascular Center (Division of Hypertension and Nephrology), Fumiki Yoshihara
・Osaka City University Graduate School of Medicine (Department of Nephrology), Katsuhito Mori, Akihiro Tsuda, Shinya Nakatani
・Osaka City General Hospital (Division of Nephrology and Hypertension), Yoshio Konishi, Takashi Morikawa, Chizuko Kitabayashi
・Osaka City General Hospital (Division of Pediatrics), Rika Fujimaru
・Osaka General Medical Center (Department of Kidney Disease and Hypertension), Terumasa Hayashi, Tatsuya Shoji
・Osaka Women's and Children's Hospital (Department of Pediatric Nephrology and Metabolism), Katsusuke Yamamoto
・Osaka Medical College (Department of Pediatrics), Akira Ashida
・Osaka Red Cross Hospital (Department of Nephrology), Akira Sugawara, Masao Koshikawa, Yoshihisa Ogawa, Tomoko Kawanishi
・Osaka Rosai Hospital (Department of Nephrology), Atsushi Yamauchi, Katsuyuki Nagatoya, Daisuke Mori, Ryota Haga
・Osaka University Graduate School of Medicine (Department of Nephrology), Yoshitaka Isaka, Ryohei Yamamoto, Tomoko Namba
・Saiseikai Shiga Hospital (Division of Nephrology), Toshiki Nishio
・Shiga University of Medical Science (Department of Medicine), Shinichi Araki
・Shirasagi Hospital (Kidney Center), Shigeichi Shoji, Kenjiro Yamakawa, Senji Okuno
・Toyonaka Municipal Hospital (Division of Nephrology), Megumu Fukunaga
・Wakayama Medical University (Department of Pediatrics), Yuko Shima, Taketsugu Hama
・Wakayama Medical University (Department of Nephrology), Takashi Shigematsu, Masaki Ohya

Chugoku District

・Kawasaki Medical School (Department of Nephrology and Hypertension), Naoki Kashihara, Tamaki Sasaki, Hajime Nagasu
・Kurashiki Central Hospital (Division of Nephrology), Kenichiro Asano, Motoko Kanzaki, Kosuke Fukuoka
・Hiroshima University Hospital (Department of Nephrology), Takao Masaki, Shigehiro Doi, Ayumu Nakashima, Toshiki Doi
・Mizushima Kyodo Hospital (Department of Nephrology), Kan Yamazaki, Nobuyoshi Sugiyama, Yuichiro Inaba, Kouji Ozeki
・Okayama Saiseikai General Hospital (Department of Nephrology), Makoto Hiramatsu, Keisuke Maruyama, Noriya Momoki
・Okayama University Graduate School of Medicine, Dentistry, and Pharmaceutical Sciences (Department of Nephrology, Rheumatology, Endocrinology and Metabolism), Hiroshi Morinaga, Ayu Akiyama, Natsumi Uchiyama, Mariko Nishiwaki
・Saiseikai Yamaguchi General Hospital (Department of Internal Medicine), Tsuyoshi Imai
・Shimane University Faculty of Medicine (Division of Nephrology), Takafumi Ito, Masahiro Egawa, Shohei Fukunaga
・Tottori University, Faculty of Medicine (Division of Pediatrics and Perinatology), Shinichi Okada, Koichi Kitamoto, Hiroki Yokoyama, Yuko Yamada

Shikoku District

・Kagawa University, Faculty of Medicine (Department of Cardiorenal and Cerebrovascular Medicine & Department of Clinical Pathology), Tadashi Sofue, Tetsuo Minamino, Emi Ibuki

・Kochi University, Kochi Medical School (Department of Endocrinology, Metabolism and Nephrology), Yoshio Terada, Taro Horino, Yoshiko Shimamura, Tatsuki Matsumoto

・Kochi University, Kochi Medical School (Department of Pediatrics), Mikiya Fujieda, Masayuki Ishihara

・Tokushima University Graduate School (Department of Pediatrics, Institute of Biomedical Sciences), Shoji Kagami, Maki Urushihara, Yukiko Kinoshita

・Tokushima University Graduate School (Department of Nephrology, Institute of Biomedical Sciences), Hideharu Abe, Kojiro Nagai

Kyushu District

・Fukuoka University (Division of Nephrology and Rheumatology, Department of Internal Medicine, Faculty of Medicine), Kosuke Masutani, Tetsuhiko Yasuno, Kenji Ito

・Japanese Red Cross Fukuoka Hospital (Department of Pediatrics), Ken Hatae, Manao Nishimura, Hiroyo Maruyama

・Japanese Red Cross Fukuoka Hospital (Nephrology and Dialysis Center), Koji Mitsuiki

・Kumamoto University Graduate School of Medical Sciences (Department of Nephrology), Masashi Mukoyama, Masataka Adachi

・Kurume University School of Medicine (Division of Nephrology, Department of Medicine), Kei Fukami, Junko Yano

・Kyushu University Graduate School of Medical Sciences (Department of Medicine and Clinical Science), Toshiaki Nakano, Akihiro Tsuchimoto, Shunsuke Yamada, Yuta Matsukuma

・Kyushu University Graduate School of Medical Sciences (Department of Environmental Medicine), Yutaka Kiyohara, Toshiharu Ninomiya, Masaharu Nagata

・Miyazaki Prefectural Miyazaki Hospital (Division of Nephrology), Naoko Yokota-Ikeda, Keiko Kodama

・Nagasaki University Hospital (Department of Pathology), Takashi Taguchi

・Nagasaki University Hospital (Department of Nephrology), Tomoya Nishino, Yoko Obata, Tadashi Uramatsu

・National Fukuoka Higashi Medical Center (Kidney Unit), Ritsuko Katafuchi

・National Hospital Organization Kyushu Medical Center, Masaru Nakayama

・Oitaken Kouseiren Tsurumi Hospital (Division of Nephrology), Ryokichi Yasumori

・Saga University, Faculty of Medicine (Department of Internal Medicine), Motoaki Miyazono, Eriko Nonaka, Shuichi Rikitake, Makoto Fukuda

・St. Mary's Hospital, Harumichi Higashi

・University of Miyazaki Hospital (Division of Nephrology), Shouichi Fujimoto, Yuji Sato, Masao Kikuchi, Akihiro Minakawa

・University of Occupational and Environmental Health (Second Department of Internal Medicine), Masahito Tamura, Tetsu Miyamoto

・University of the Ryukyus Graduate School of Medicine (Department of Cardiology, Nephrology and Neurology), Yusuke Ohya, Kentaro Kohagura
